# Supplementary material for: Pathogenic Escherichia coli Possess Elevated Growth Rates under Exposure to Sub-Inhibitory Concentrations of Azithromycin
Source: Antibiotics (Basel). 2020 Oct 26;9(11):735. doi: 10.3390/antibiotics9110735 (PMC7693856; doi:10.3390/antibiotics9110735)
Supplement: Supplementary file 1 [file antibiotics-09-00735-s001.pdf]

Supplementary Materials

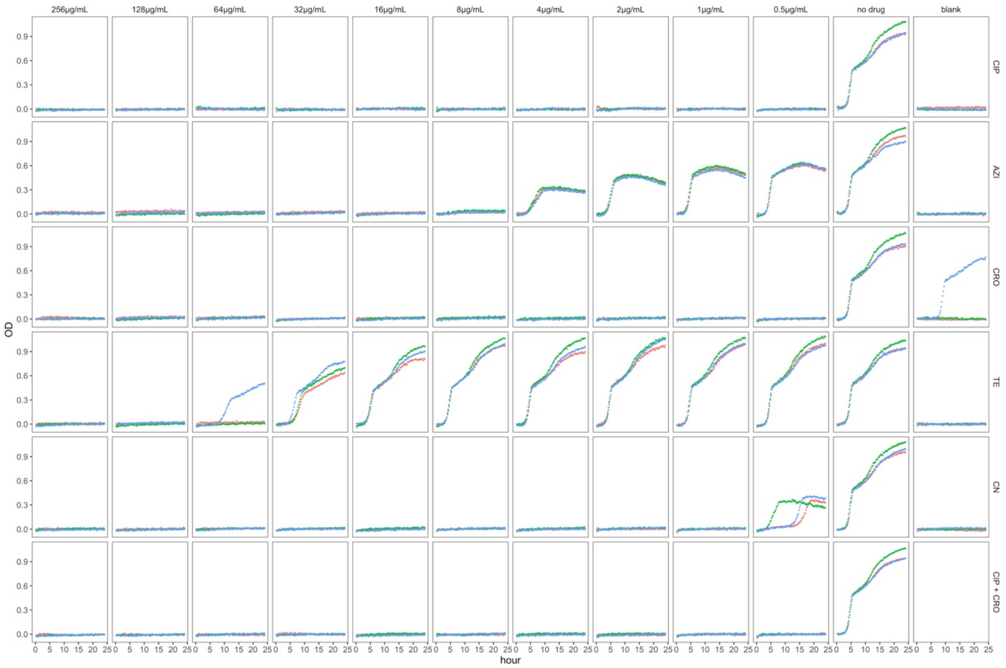

Figure S1. Three replicates of growth dynamics monitoring**.** A clinical *E. coli* isolate was treated, three times, with ciprofloxacin (CIP), azithromycin (AZI), ceftriaxone (CRO), tetracycline (TE), and gentamicin (CN) at different concentrations. Red dots: replicate #1, green dots: replicate #2, and blue dots: replicate #3.

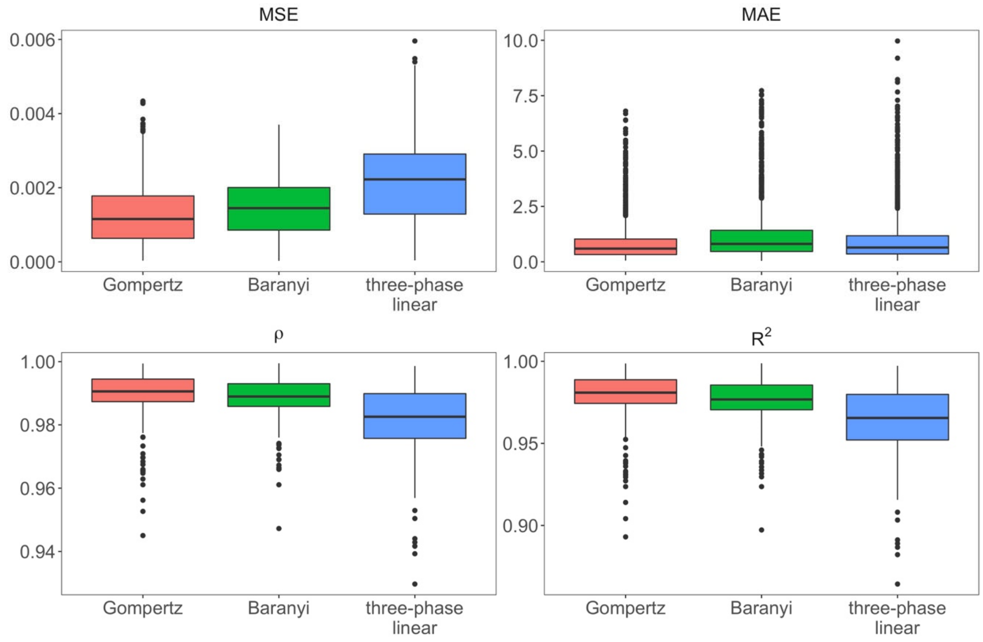

Figure S2. Comparison between performance of Gompertz's, Baranyi's and three-phase linear model**.** For each growth curve, the three models were utilized to predict OD values at every timepoints from 0 to 24h. The actual and predicted values were used to calculate the mean square error (MSE), mean absolute error (MAE), correlation coefficient (ρ), and correlation of determination (R^2). The lower MSE and MAE; higher ρ and R^2, the better a model is.

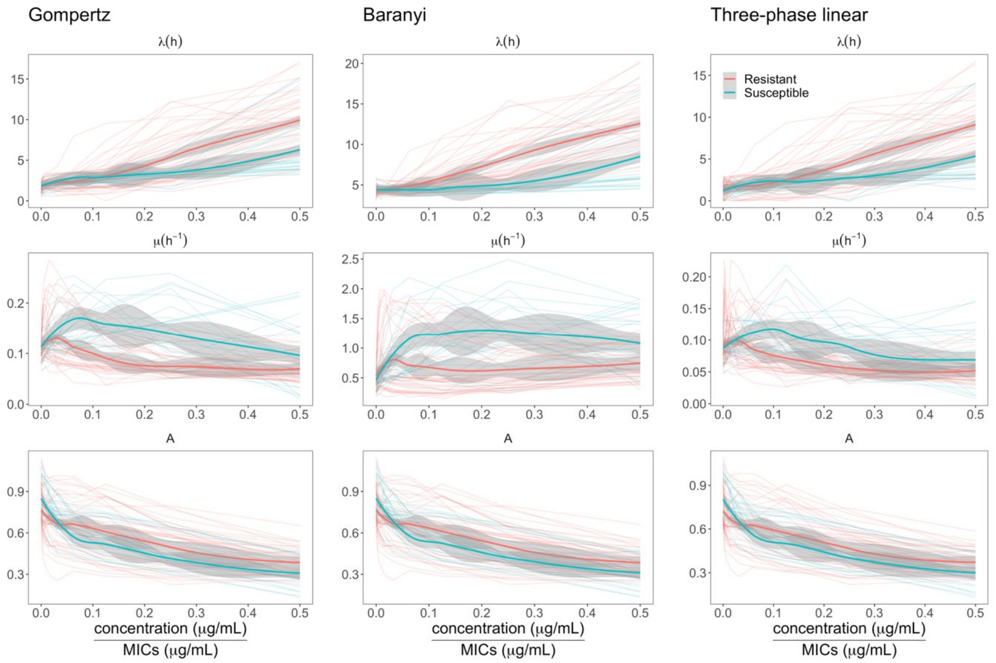

Figure S3. Comparison between growth parameters estimated from Gompertz's, Baranyi's and three-phase linear model**.** Parameters include lag-phase period (λ), maximum growth rate (μ), and maximum cell density (A). The horizontal and vertical axes are relative concentration of antimicrobials and values of the growth parameters, respectively.
